# HSP90 N‐terminal inhibitors target oncoprotein MORC2 for autophagic degradation and suppress MORC2‐driven breast cancer progression

**DOI:** 10.1002/ctm2.825

**Published:** 2022-05-06

**Authors:** Fan Yang, Rui Sun, Zeng Hou, Fang‐Lin Zhang, Yi Xiao, Yun‐Song Yang, Shao‐Ying Yang, Yi‐Fan Xie, Ying‐Ying Liu, Cheng Luo, Guang‐Yu Liu, Zhi‐Min Shao, Da‐Qiang Li

**Affiliations:** ^1^ Fudan University Shanghai Cancer Center and Shanghai Key Laboratory of Medical Epigenetics, International Co‐laboratory of Medical Epigenetics and Metabolism, Ministry of Science and Technology, Institutes of Biomedical Sciences Fudan University Shanghai China; ^2^ Cancer Institute Shanghai Medical College, Fudan University Shanghai China; ^3^ Department of Oncology Shanghai Medical College, Fudan University Shanghai China; ^4^ Department of Breast Surgery Shanghai Medical College, Fudan University Shanghai China; ^5^ School of Pharmaceutical Science and Technology Hangzhou Institute for Advanced Study, UCAS Hangzhou China; ^6^ Drug Discovery and Design Center, The Center for Chemical Biology, State Key Laboratory of Drug Research, Shanghai Institute of Materia Medica Chinese Academy of Sciences Shanghai China; ^7^ Department of Pharmacy University of Chinese Academy of Sciences Beijing China; ^8^ Shanghai Key Laboratory of Breast Cancer Shanghai Medical College, Fudan University Shanghai China; ^9^ Shanghai Key Laboratory of Radiation Oncology Shanghai Medical College, Fudan University Shanghai China

**Keywords:** breast cancer, chaperone‐mediated autophagy, HSP90 inhibitor, MORC2, protein degradation

## Abstract

**Aims:**

MORC family CW‐type zinc finger 2 (MORC2), a GHKL‐type ATPase, is aberrantly upregulated in multiple types of human tumors with profound effects on cancer aggressiveness, therapeutic resistance, and clinical outcome, thus making it an attractive drug target for anticancer therapy. However, the antagonists of MORC2 have not yet been documented.

**Methods and Results:**

We report that MORC2 is a relatively stable protein, and the N‐terminal homodimerization but not ATP binding and hydrolysis is crucial for its stability through immunoblotting analysis and Quantitative real‐time PCR. The N‐terminal but not C‐terminal inhibitors of heat shock protein 90 (HSP90) destabilize MORC2 in multiple cancer cell lines, and strikingly, this process is independent on HSP90. Mechanistical investigations revealed that HSP90 N‐terminal inhibitors disrupt MORC2 homodimer formation without affecting its ATPase activities, and promote its lysosomal degradation through the chaperone‐mediated autophagy pathway. Consequently, HSP90 inhibitor 17‐AAG effectively blocks the growth and metastatic potential of MORC2‐expressing breast cancer cells both in vitro and in vivo, and these noted effects are not due to HSP90 inhibition.

**Conclusion:**

We uncover a previously unknown role for HSP90 N‐terminal inhibitors in promoting MORC2 degradation in a HSP90‐indepentent manner and support the potential application of these inhibitors for treating MORC2‐overexpressing tumors, even those with low or absent HSP90 expression. These results also provide new clue for further design of novel small‐molecule inhibitors of MORC2 for anticancer therapeutic application.

## Graphical abstract

- HSP90 N‐terminal inhibitors promote autophagic degradation of MORC2
- MORC2 degradation induced by HSP90 N‐terminal inhibitors is independent on HSP90
- HSP90 N‐terminal inhibitors disrupt MORC2 homodimerization to induce its degradation
- HSP90 N‐terminal inhibitors suppress MORC2‐mediated breast cancer progression both in vitro and in vivo

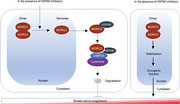

## 1 INTRODUCTION

Chromatin‐remodelling proteins play fundamental roles in almost all DNA‐related biological processes, through modulating nucleosome positions and local chromatin structure.^1^, ^2^ Consequently, their dysregulation contributes to various human diseases including cancer. One of such proteins is MORC family CW‐type zinc finger 2 (MORC2), a protein of the GHKL (*g*yrase, *h*eat shock protein 90, histidine *k*inase and Mut*L*) superfamily which also contains DNA topoisomerase II (TOP2), heat shock protein 90 (HSP90), histidine kinases, DNA mismatch repair enzyme MutL and epigenetic factor SMCHD1.^3^, ^4^ All of these enzymes utilize a common Bergerat fold to bind ATP, but exhibit divergent protein structures and biological functions.^3^ Emerging structural and biochemical evidence shows that MORC2 contains a catalytically active ATPase module, a CW‐type zinc finger (CW‐ZF) domain, a chomo‐like domain and three coiled‐coil (CC) domains.^4^, ^5^, ^6^ The N‐terminal module of MORC2 is composed of a GHKL‐type ATPase and a S5‐fold domain (recently named as transducer‐like domain) with the insertion of a CC domain (CC1) into the S5 domain.^6^ Upon ATP binding, the ATPase module of MORC2 N‐terminus dimerizes and becomes active to alter chromatin architecture at heterochromatic target sites occupied by the Human Silencing Hub (HUSH) complex, thus regulating the gene transcription.^5^, ^6^, ^7^ In addition, MORC2 exerts an emerging chromatin remodelling activity to regulate cellular response to DNA damage.^8^

The functional importance of MORC2 is highlighted by two lines of evidence. First, MORC2 is aberrantly upregulated in multiple types of tumours and promotes aggressive and therapeutically resistant phenotypes.^9^, ^10^, ^11^, ^12^ The systematic work from our group recently shows that MORC2 is of importance in breast cancer progression. Moreover, MORC2 contributes to resistance to endocrine therapy and chemoresistance.^13^, ^14^, ^15^, ^16^ Second, the ATPase module mutations of MORC2 are related in the progression of refractory breast cancer^16^ and pathogenesis of axonal Charcot‐Marie‐Tooth (CMT) disease^17^, ^18^, ^19^ and neurodevelopmental disorder.^20^ These exciting findings have aroused wide interest in identifying MORC2 antagonists to combat these MORC2‐related diseases. However, the drugs targeting this protein have not yet been reported.

Over the past decades, two major strategies have been developed to target specific oncoproteins, including monoclonal antibodies (mAbs) and small‐molecule inhibitors. As an antigen that can be effectively targeted by a specific mAb needs to be expressed on the surface of cancer cells,^21^ MORC2 is not an ideal target antigen to develop antibody‐based therapeutics because it is predominantly localized in the nucleus.^8^, ^13^, ^15^, ^16^ Given that MORC2 has enzymatic activities and forms a homodimer,^5^, ^6^, ^8^ we first sought to identify its small‐molecule inhibitors through repurposing existing regimens in light of increasing failure rates and the high cost of the improvement of new molecularly targeted drugs.

HSP90, a molecular chaperone, exists in the form of a homodimer. Each monomer is composed of three functional domains, including a N‐terminal domain (NTD) responsible for ATP binding and hydrolysis, a middle domain and a C‐terminal domain (CTD) essential for dimerization.^22^ It has been well documented that HSP90 is of primary importance in inducing and replicating a variety of biogenic signalling proteins, and many of them are oncoproteins such as Akt, cyclin D1 and cylin‐dependent kinase 4 (CDK4).^23^ Consequently, pharmacological inhibition of HSP90 abolishes HSP90‐dependent folding of immature client proteins and directs them to proteolysis.^24^, ^25^ To date, dozens of HSP90 inhibitors have been developed through targeting the NTD or CTD of HSP90. In this context, the N‐terminal inhibitors, such as AUY922 (Luminespib),17‐AAG (Tanespimycin) and STA‐9090 (Ganetespib), bind to the ATP‐binding pocket of HSP90 and consequently suppress ATP‐dependent chaperone activity.^26^ In contrast, C‐terminal inhibitors of HSP90 chaperone, such as novobiocin (NB) and its analogues, bind to the C‐terminus and induce its conformational changes to release client proteins.^27^ In addition, epilgallocatechin‐3‐gallate (EGCG), the important constituent of green tea, and cisplatin, a platinum‐containing chemotherapeutic drug, have been shown its interaction with the HSP90 C‐terminus and decrease molecular chaperone activity.^26^, ^27^ Currently, more than 20 clinical trials for the treatment of HSP90‐targeted agents for cancer therapy are underway, but clinical efficacy is highly variable in different settings.^23^ Thus, selection of suitable patients who may benefit from treatment with HSP90 inhibitors through identifying predictive biomarkers for the efficacy of these agents is most important towards the goal of optimizing the use of these agents in the clinic.

Here, we provide the first evidence that HSP90 N‐terminal but not C‐terminal inhibitors promote lysosomal degradation of MORC2 through disrupting its homodimerization, and this process is HSP90 independent. Moreover, HSP90 N‐terminal inhibitor 17‐AAG is effective against MORC2‐expressing breast tumours, and the noted effect is not due to inhibition of HSP90.

## 2 MATERIALS

### 2.1 Cell culture and chemicals

Cancer cell lines, MCF‐7, LM2‐4175, T47D, JIMT‐1, SK‐BR‐3, MDA‐MB‐231, Hs578T (breast cancer), HeLa (cervical cancer), MHCC97‐L (liver cancer), A549 (lung cancer), ES‐2 (ovarian cancer), MIApaca‐2 (pancreatic cancer) and human embryonic kidney 293T (HEK293T) cell line, were collected from the Type Culture Collection of the Chinese Academy of Sciences (Shanghai, China). All cell lines were authenticated by monitoring cell vitality, mycoplasma contamination and short tandem repeat profiling. After receiving those cancer cells in 2014, the cells were distended and frozen presently into many equal parts. McCoy's 5A (BasalMedia, #L630) was used to culture SK‐BR‐3 cell. High‐glucose DMEM medium (BasalMedia, #L110) was used to maintain the other cell lines. All medium was contained with 1% penicillin‐streptomycin (BasalMedia, #S110B) plus 10% foetal bovine serum (ExCell Biol, #FSP500). See Table S1 for details of the chemical inhibitors used in this study.

### 2.2 DNA constructs, transfection and viral transduction

Full‐length Flag‐MORC2 and HA‐MORC2 constructs have been described previously.^28^ PCR‐based methods were used for generating the site‐directed mutagenesis （all primers in Table S2 in details). Small interfering RNA (siRNA) of HSPA8, LAMP2A, non‐targeting negative control (siNC) were obtained from GenePharma (Shanghai, China) (see Table S3 for details). Short hairpin RNAs (shRNAs) silencing human MORC2 (shMORC2) in lentiviral pGFP‐C‐shLenti vector (#TL311427) were obtained from Origene. All constructs were proved by oligonucleotide array sequence analysis. LentiGuide‐Puro (Addgene, #52963) and lentiCas9‐Blast (Addgene, #52962) plasmids were from Feng Zhang laboratory. CRISPR/Cas9 system was used to construct the HSP90 knockout (KO) cell lines.^29^ The HSP90 KO cell lines were validated in protein level and further subjected to Sanger sequencing. See Table S4 for individual gRNA sequences. Transient plasmid transfection was carried out using Neofect DNA transfection reagent (Tengyi Biotech, #TF201201). In order to establish breast cancer cell lines stably expressing sgRNA, shRNA or cDNAs, lentivirus expression vector and packaging plasmid mix were used to transfect HEK293T cells. In addition, the collected supernatant was used for infecting target cells for 48 h prior to drug selection (2 μg/ml of puromycin, Sangon Biotech, #A610593‐0025) for 1 week. Lipofectamine 2000 Transfection Reagent (Invitrogen, #11668019) was used for siRNA transfection.

### 2.3 Immunoblotting, immunoprecipitation and antibodies

For immunoblotting analysis, cells were lysed in modified RIPA buffer (1% Nonidet P‐40, 50 mM Tris‐HCl, pH7.4, 0.25% sodium deoxycholate, 150 mM NaCl and 1 mM EDTA, 0.1% sodium dodecyl sulfate) added with phosphatase inhibitors and protease inhibitors (Bimake, #B14002 and #B15003, respectively). Cellular extracts were separated by SDS‐PAGE, and transferred to PVDF membranes (Millipore, #IPVH00010). Membranes were incubated with the indicated primary antibodies and detected. To immunoprecipitate exogenous and endogenous proteins, cell extracts were incubated with primary antibodies or control IgG for 6 h at 4°C, and extra 2 h with additional protein A/G magnetic beads (Bimake, #B23202), then washed and subjected to immunoblotting analysis. The quantitation of immunoblotting bands was performed by ImageJ software (National Institutes of Health, USA). See Table S5 for details information of primary antibodies. All of secondary antibodies were obtained from Cell Signaling Technology.

### 2.4 Quantitative real‐time PCR

TRIzol reagent (Invitrogen, #15596018) were used to isolate the cellular RNA. Then, total RNA of indicated cells were transcribed into cDNA using PrimeScript RT Master Mix (Vazyme, #R323‐01). Quantitative real‐time PCR (qPCR) was carried out using ChamQ Universal SYBR qPCR Master Mix (Vazyme, #Q711‐03) on an Eppendorf Mastercycler ep realplex4 instrument. Gene expression levels were normalized against β‐actin using the relative 2^–ΔΔCt^ method and are showed as relative expression compared to the control. See Table S6 for all primers.

### 2.5 Molecular modelling and molecular docking studies

The crystal structure of HSP90 N‐terminal (PDB: 2YEF) and the crystal structure of MORC2 N‐terminal (PDB: 5OF9) were downloaded from the Protein Data Bank. All of the solvent molecules were removed. The comparison of the two proteins crystal structures was generated using PyMOL version 2.0.

To explore the binding modes of these three HSP90 compounds to MORC2, we conducted molecular docking studies. Firstly, the protein structure was prepared using Protein Preparation Wizard module in the Maestro version 11.1 to optimize the protein status with the pH value of 7.4 ± 0.0. In the optimization process, all parameters were set by default. Next, the Ligprep module in the Maestro program was used to generate 3D coordinates of the three ligands in default mode. Finally, the prepared protein and ligands were docked under the Induced Fit Docking module in the Maestro. All of the binding‐mode figures were generated using PyMOL version 2.0.

### 2.6 Protein expression and purification

The DNA fragment coding the human MORC2 N‐terminal (residues 1–282) was cloned into a pET15b vector for His‐tagged N‐terminal protein and expressed in *Escherichia coli* BL21 (DE3) cells. The 2×YT media was used to culture the cells for 4–6 h until OD600 reached 0.7–0.8 at 37°C. Then, the cells were induced with 0.2 mM isopropyl β‐D‐thiogalactopyranoside for 18 h at 18°C to express the target protein. The cells were harvested by centrifugation at 2500 rpm for 40 min and resuspended in the pre‐cooled lysis buffer (50 mM Tris‐HCl, pH 8.0, 500 mM NaCl, 1 mM DTT and 1× complete EDTA‐free protease inhibitors [Roche]). The supernatant was collected after the cells were lysed and centrifuged. The 6×His‐tagged fusion protein was purified through HisTrap FF column (GE Healthcare) with elution buffer (50 mM Tris‐HCl pH 8.0, 500 mM NaCl, 1 mM DTT, 100 mM imidazole). Then, fusion protein was purified through a Mono Q column (GE Healthcare) after condition in which the protein present was changed from elution buffer to QA buffer (50 mM Tris‐HCl, pH 8.0, 1 mM DTT), followed by gel‐filtration chromatography on a Superdex 200 increase (10/300) column (GE Healthcare). In the end, the purified human MORC2 N‐terminal (residues 1–282) protein was stored in buffer containing 50 mM HEPES (pH 7.5), 150 mM NaCl, 2 mM MgCl_2_ and 0.25 mM TCEP at −80°C.

### 2.7 Thermal shift assay

Thermal shift assays (TSAs) were performed on a real‐time PCR instrument (QuantStudio 6 Flex, Applied Biosystems). 5× SYPRO Orange dye (ThermoFisher Scientific) was mixed with the final concentration of 10 μM MORC2 N‐terminal protein and a series of diluted compounds in the TSA buffer (50 mM HEPES, pH 7.5, 150 mM NaCl, 2 mM MgCl_2_, 0.25 mM TCEP). Twenty microlitres of reaction mixture per well were loaded in Optical 96‐well reaction plate (Applied Biosystems). The mixture was heated up by 0.05°C/s from 25°C to 95°C and tested in triplicate. The monitored fluorescence signal from the SYPRO orange dye was applied to evaluate the melting temperature (Tm) of each sample with Protein Thermal Shift software v1.3 (ThermoFisher Scientific).

### 2.8 Half‐life assays of proteins

In order to estimate the half‐life of proteins, indicated cells were cultured with 100 μg/ml cycloheximide (CHX) (Cell Signaling Technology, #2112S) after transfection, followed by collection of cells at indicated time points and then subjected to immunoblotting analysis.

### 2.9 Glutaraldehyde cross‐linking assays

Cells were lysed with the NP40 buffer and crosslinked with 0.05% (w/v) glutaraldehyde (Sigma, #G6257‐100ML) on ice for 5 min. After that, the cross‐link reaction was terminated by 1 M glycine for 15 min at room temperature. The samples were subjected to immunoblotting analysis by 8% SDS‐PAGE.

### 2.10 ATPase assays

ATPase activities of MORC2 were assessed using an ATPase/GTPase assay kit (Sigma, #MAK113) following the manufacturer's protocol. We immunoprecipitate exogenously expressed Flag‐MORC2 according the immunoprecipitation protocols. After that, 30 μl of Reaction Mixes containing 20 μl assay buffer and 10 μl 4 mM ATP (Sigma, #A1852‐1VL) was supplemented into the tubes with immunoprecipitated Flag‐MORC2 beads in the presence of DMSO, 1 μM 17‐AAG, 1 μM AUY922, 1 μM STA‐9090 or 10 μM NB. After incubation at room temperature for about 1 h, 200 μl of reagent was added and incubated for extra 30 min. At once, the absorbance at 620 nm was read and analysed.

### 2.11 Cell viability, colony formation, cell migration and invasion assays

To test the cell viability, a total of 3 ×10^3^ cells were plated into 96‐well plate cells. The following day, 100 μl of fresh medium containing the corresponding concentration of various inhibitors was added to each well to replace the growth medium for 72 h drug exposure. Cell Counting Kit‐8 (Yeasen, #40203ES92) was used to estimate the cell inhibition with the absorbance at 450 nm (A450). Three‐parameter logistic curve fitting method was used to analyse the concentration of drug resulting in 50% inhibition of cell viability (IC_50_). For colony formation survival assays, a total of 5 ×10^3^ cells were plated into 12‐well plate and treated with indicated drugs. The medium containing inhibitors replaced every 3 days. After 14 days of treatment, cells were fixed in methanol and stained with 0.5% crystal violet. For Transwell migration and invasion (Matrigel) assays, 3×10^4^ cells in serum‐free media were plated in the upper chambers (Corning BioCoat, #354480, Corning Falcon, #353097, respectively). After 24–48 h, cells in the lower membranes of transwell chambers were fixed and stained with 0.5% crystal violet.

### 2.12 Immunofluorescent staining

4% Methanol‐free formaldehyde (Yeasen, #36314ES76) were used to fix the cells after treatment. After that, cells were permeabilized with 0.5% Triton X‐100 for 20 min at 4°C. Cells were blocked with 5% goat serum for 1 h and then incubated with anti‐HSPA8 (1:200), anti‐LAMP2A (1:200) and anti‐Flag (1:500) antibody in 5% goat serum overnight at 4°C and incubated with the corresponding secondary antibodies conjugated with Alexa‐488 or Alexa‐555 (1:500) at room temperature for 1 h. Afterwards, the cells were sealed with a DAPI‐containing fluoroshield mounting medium (Abcam, #ab104139). Leica SP5 microscope was used to visualize the images, and ImageJ software was used for the correlation analysis.

### 2.13 In vivo lung metastasis assays

All animal experiment protocols were reviewed and approved by the Institutional Animal Care and Use Committee at Fudan University. For subcutaneous inoculation, 1.5×10^6^ LM2‐4175 parental cells and LM2‐4175 MORC2 knockout (KO) cells were injected subcutaneously into the tail veins of BALB/c female nude mice (Shanghai SLAC Laboratory Animal Co., Ltd). One week after injection, animals were randomly assigned to one of three groups and were treated with vehicle control or 17‐AAG 60 mg/kg by peritoneal injection (continuous treatment for 5 days, interval of 2 days and a total of 3 weeks) following the protocol described previously.^30^ 17‐AAG was predissolved in DMSO and further diluted in corn oil. One week after treatment, mice were euthanized, and the lungs were removed from the mice and metastatic nodules of lungs were counted.

### 2.14 Immunohistochemical staining

Lung specimens obtained from mice were fixed with 10% formalin and then subjected to immunohistochemical (IHC) staining with an anti‐MORC2 (Novus, NBP1‐89295, 1:50) or anti‐cyclin D1 (Cell Signaling Technology, #2978S, 1:150) antibody. Olympus BX43 microscope was used to take the representative photographs. ImageJ software was used for the quantifications of all IHC staining.

### 2.15 Cytosol‐nucleus cell fractionation assay

Cells were collected into 1 ml of ice‐cold PBS containing 1 mM PMSF after 48 h of transfection and subjected to centrifuge processing. After removing the supernatant, cells were resuspended with 1 ml of buffer A (300 mM sucrose, 20 mM HEPES, pH 7.9, 10 mM KCl, 1.5 mM MgCl_2_, 1 mM dithiothreitol). Then, cells were rotated for 15 min at 4℃ and added with 1 ml of buffer B (buffer 1 supplemented with 0.5% Nonidet P40) and rotated for 10 min 4℃. The cytoplasmic fraction was collected after subjecting to centrifuge processing at 23,000 rpm for 20 min. Then, cells were re‐suspended with buffer C (25% Glycerol, 20 mM pH 7.9 HEPES, 420 mM KCl, 0.5 mM DTT,1.5 mM MgCl_2_, 0.2 mM EDTA) and rotated for 1–2 h at 4℃. Nucleus fraction was collected after spinned down at 15,000 rpm for 30 min.

### 2.16 Statistical analysis

All data are showed as the mean ± standard error (mean ± SD) from at least three independent experiments. Volcano plots of the RNA‐seq data from Gene Expression Omnibus (GEO) dataset GSE95452^31^ were generated using R with the EnhancedVolcano package (version 4.0.2). The Student's *t*‐test by Graphpad Prism 9.0 was used calculate the difference between groups. *p* Values below .05 were considered as statistically significance.

## 3 RESULTS

### 3.1 HSP90 N‐terminal, but not C‐terminal, inhibitors destabilize MORC2 in multiple cancer cell lines

**FIGURE 1:**
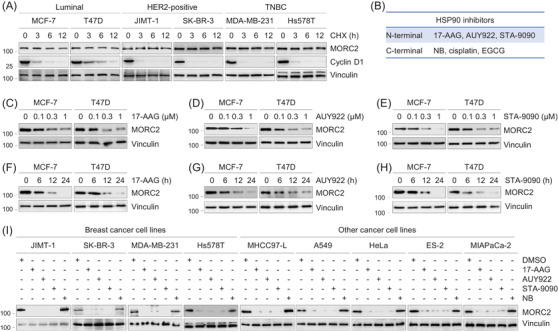
HSP90 N‐terminal, but not C‐terminal, inhibitors downregulate MORC2 in multiple cancer cell lines. (A) Cells were treated with 100 μg/ml of CHX for 0, 3, 6 and 12 h, and total cellular lysates were harvested for immunoblotting analysis with the indicated antibodies. (B) Six HSP90 inhibitors used in this study. (C–E) MCF‐7 and T47D cells were treated with or without 17‐AAG (C), AUY922 (D) and STA‐9090 (E) at the indicated doses for 24 h. Total cellular lysates were harvested for immunoblotting analysis with the indicated antibodies. (F–H) MCF‐7 and T47D cells were treated with or without 1 μM of 17‐AAG (F), AUY922 (G) and STA‐9090 (H) for the indicated times. Total cellular lysates were harvested for immunoblotting analysis with the indicated antibodies. (I) Nine different cancer cell lines were treated with DMSO or 1 μM of 17‐AAG, AUY922, STA‐9090 or 10 μM of NB for 24 h. Total cellular lysates were harvested for immunoblotting analysis with the indicated antibodies

MORC2 has been documented to be overexpressed in 15 types of common human tumours including breast cancer.^9^ Clinically, breast cancer is divided into three major molecular subtypes with distinguishing biological function and distinct clinical outcome, including luminal, HER2‐positive and triple‐negative breast cancer (TNBC). In order to understand the internal regulatory mechanism of MORC2, we carried out CHX chase assays to determine its turnover rate in six commonly used cell lines of three distinguishing breast cancer molecular subtypes. As displayed in Figure 1A, in contrast to cell‐cycle regulator Cyclin D1, a known labile protein with a half‐life of less than 30 min,^32^ MORC2 remained relatively stable even after 12 h of CHX treatment. Human MORC2 pre‐mRNA generates 2 transcript variants (NM_001303256 and NM_014941), which encode two protein isoforms, including full‐length MORC2 (residues 1–1032; UniProt: Q9Y6 × 9‐1) and a truncated mutant lacking the first 62 amino acids at the N‐terminus (∆62) (residues 63–1032; UniProt: Q9Y6 × 9‐2), respectively.^18^ CHX chase assays showed that full‐length MORC2 was more stable than ∆62 mutant one (Figure S1a,b). These results indicate that intact N‐terminal ATPase module of MORC2 is crucial for its stability.

As MORC2 and HSP90 have a similar Bergerat ATP‐binding fold,^3^ we next investigated whether HSP90 inhibitors have any effects on MORC2 stability. Towards this aim, we first treated luminal‐type MCF‐7 and T47D breast cancer cells with three HSP90 N‐terminal inhibitors (17‐AAG, AUY922 and STA‐9090) and three HSP90 C‐terminal inhibitors (NB, cisplatin and EGCG) (Figure 1B and Tables S7 and S8).^26^, ^27^ These inhibitors have entered phase I/II clinical trials, in addition to first‐line chemotherapy drug cisplatin in breast cancer (Supplementary Table S7). Immunoblotting analysis showed a decrease of MORC2 in the protein levels following three N‐terminal inhibitors treatment (Figure 1C–H). Three HSP90 C‐terminal inhibitors (NB, cisplatin and EGCG)^26^, ^27^ had no remarkable effects on MORC2 protein levels (Figure S2a–f). These data reveal that the N‐terminal, but not C‐terminal, inhibitors of HSP90 downregulate MORC2. Analogous results were also obtained in additional nine cancer cell lines, including MDA‐MB‐231, Hs578T (TNBC), JIMT‐1, SK‐BR‐3 (HER2‐positive), MHCC97‐L (liver cancer), A549 (lung cancer), HeLa (cervical cancer), ES‐2 (ovarian cancer), and MiaPaCa‐2 (pancreatic cancer) (Figure 1I), highlighting that the effects of HSP90 N‐terminal inhibitors on MORC2 downregulation are not cell‐line specific.

Human DNA topoisomerase II (TOP2), another important target in anticancer therapy, is also a member of the GHKL superfamily.^3^ As NB is not only a HSP90 C‐terminal inhibitor, but also an eukaryotic TOP2 inhibitor by blocking the ATP‐binding site,^27^ we next tested the effects of another two TOP2 ATPase inhibitors, O6‐benzylguanine (O6‐BG) and its analogue NU2058,^33^ on MORC2 expression levels. As displayed in Figure S2g, treatment with either of those two drugs could not remarkably alter the expression levels of MORC2. We further showed that adriamycin (doxorubicin), a TOP2 poison by stabilizing the TOP2‐DNA covalent complex, has no evidential effects on MORC2 protein levels in breast cancer cells.^13^ These results indicate that TOP2 inhibitors have no remarkable effects on MORC2 expression levels.

### 3.2 HSP90 N‐terminal inhibitors have potential binding pockets in MORC2

In order to explore the molecular basis of those three shown inhibitory activities against both HSP90 N‐terminal inhibitors and MORC2, we compared the ATP binding pocket of HSP90 with MORC2. Firstly, we aligned the crystal structure of HSP90 N‐terminal (PDB: 2YEF) with the crystal structure of MORC2 N‐terminal (PDB: 5OF9), the results show that the helix2 (H2), helix3 (H3), helix5 (H5) and sheet1 (S1) of HSP90 N‐terminal have high structural homology to MORC2 N‐terminal (Figure S3a). Meanwhile, the residues which are critical for recognition of Hsp90 to the nucleotide binding, asparagine 51 (N51), aspartic acid 93 (D93), glycine 97 (G97), threonine 184 (T184), are highly homologous to asparagine 39 (N39), aspartic acid 68 (D68), glycine 72 (G72), threonine 197 (T197) of MORK2. In addition, those residues which form key interaction with phosphate group of ATP, glutamic acid 47 (E47), aspartic acid 54 (D54) and glycine 137 (G137), have great homology with glutamic acid 35 (E35), aspartic acid 42 (D42) and glycine 103 (G103) of MORK2. Alanine 55 (A55) and methionine 98 (M98) involved in forming the ATP pocket of HSP90 are highly homologous to alanine 43 (A43) and methionine 73 (M73) of MORC2 (Figure S3b,c). To explore the binding modes of these three HSP90 N‐terminal inhibitors, we conducted molecular docking studies. The results indicated that these three HSP90 N‐terminal inhibitors well occupied the ATP‐binding pocket (Figure S3d). To validate the direct binding of those compounds with MORC2, we performed the TSA to verify that these three compounds can directly bind to the N‐terminal of MORC2 in vitro. The inflection points of the melting curve, which corresponds to the midpoint of the protein denaturation process, is the melting temperature (Tm).Upon ligand binding, the stabilized protein exhibits a higher Tm. AUY922 and STA‐9090 significantly stabilized MORC2 N‐terminal (i.e., led to a large increase in the melting temperature, Tm, Figure S3e,f), doing so in a concentration‐dependent manner. However, the binding ability of 17‐AAG to MORC2 could not be verified by TSA due to its strong colour interference. These modelling and TSA data suggest that HSP90 N‐terminal inhibitors bind directly to the N‐terminus of MORC2.

### 3.3 HSP90 N‐terminal inhibitors regulate MORC2 at post‐transcriptional level

We next performed qPCR analysis to determine whether the noted effects of HSP90 N‐terminal inhibitors on MORC2 occur at transcriptional level. In Figures S4 and S5, the mRNA levels of MORC2 were not remarkably altered in these cancer cell lines following treatment with HSP90 inhibitors. CHX chase assays suggested that the half‐life of MORC2 protein was remarkably shortened in the presence of HSP90 N‐terminal inhibitors (17‐AAG, AUY922 and STA‐9090) (Figure S6). These results suggest that HSP90 N‐terminal inhibitors destabilize MORC2 protein in cancer cell lines.

### 3.4 HSP90 N‐terminal inhibitors affect downstream target genes of MORC2

To examine whether HSP90 N‐terminal inhibitors affect downstream target genes of MORC2, we first analysed RNA‐sequencing data from GEO dataset GSE95452, which includes RNA‐seq results of three independent knockout HeLa clones lacking MORC2 (GSM2514487) and wild‐type HeLa cells (GSM2514486).^31^ Genes that are down‐ or upregulated significantly following MORC2 knockout are shown in Figure S7a. Next, we selected two putative oncogenes MUC16 and Hes Family BHFH Transcription Factor 1 (HES1) for further validation by qPCR using wild‐type and MORC2‐depleted LM2‐4175 and BT549 cells after treating with HSP90 N‐terminal inhibitors. MUC16, also known as cancer antigen 125 (CA125), is a novel biomarker and a novel target for human cancers.^34^ In addition, HES1 is recognized as potential stocks in stem cell fate, epithelial‐mesenchymal transition process and carcinogenesis.^35^, ^36^, ^37^ Data indicated that mRNA levels of HES1 and MUC16 were remarkably reduced in wild‐type LM2‐4175 and BT549 cells, relative to MORC2‐depleted cells, after treating with HSP90 N‐terminal inhibitors (Figure S7b).

### 3.5 HSP90 N‐terminal inhibitors promote autophagic degradation of oncoprotein MORC2 through the chaperone‐mediated autophagy pathway

**FIGURE 2:**
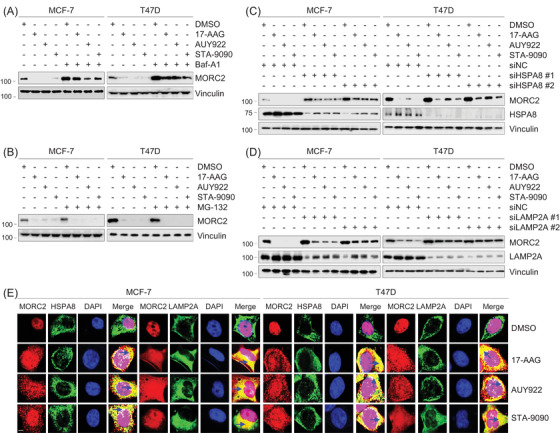
HSP90 N‐terminal inhibitors induce lysosomal degradation of MORC2 through the CMA pathway. (A) MCF‐7 and T47D cells were treated with DMSO or 1 μM of 17‐AAG, AUY922, STA‐9090 alone or in combination with 100 nM Baf A1 for 24 h. Total cellular lysates were harvested for immunoblotting analysis with the indicated antibodies. (B) MCF‐7 and T47D cells were treated with DMSO or 1 μM of 17‐AAG, AUY922, STA‐9090 alone or in combination with 10 μM MG‐132 for 24 h. Total cellular lysates were harvested for immunoblotting analysis with the indicated antibodies. (C,D) MCF‐7 and T47D cells were transfected with control siRNA (siNC) or two independent siRNAs targeting HSPA8 (siHSPA8) or LAMP2A (siLAMP2A). After 24 h of transfection, cells were treated with DMSO or 1 μM of 17‐AAG, AUY922 or STA‐9090 for 24 h. Total cellular lysates were harvested for immunoblotting analysis. (E) MCF‐7 and T47D cells were transfected with Flag‐MORC2. After 24 h of transfection, cells were treated with DMSO or 1 μM of 17‐AAG, AUY922 or STA‐9090 for 24 h, and then subjected to immunofluorescent staining with the anti‐Flag (red), anti‐HSPA8 (green) and anti‐LAMP2A (green) antibodies. DNA was counterstained with DAPI. Scale bar, 10 μm. The dark blue arrows in the merged images indicate the co‐localization of MORC2 with HSPA8 or LAMP2A in the cytoplasm (yellow colour)

There are two routes for intracellular protein degradation in eukaryotes, one of which is the ubiquitin–proteasome system and the other is the autophagy–lysosome pathway. In order to decide which pathway is attached to MORC2 degradation induced by HSP90 inhibitors, MCF‐7 and T47D cells were cultured with three HSP90 N‐terminal inhibitors alone or in combination with proteasome inhibitor MG‐132 or autophagy–lysosome inhibitor bafilomycin A1 (Baf A1). Immunoblotting analysis showed that preincubation with Baf A1 (Figure 2A), but not MG‐132 (Figure 2B), partially restored HSP90 inhibitor‐induced downregulation of MORC2. These data uncover that the autophagy–lysosome pathway is of importance in MORC2 degradation induced by HSP90 inhibitors.

**FIGURE 3:**
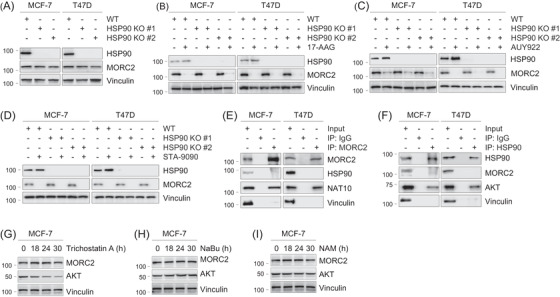
Downregulation of MORC2 by HSP90 N‐terminal inhibitors is independent on HSP90. (A) Both HSP90α and HSP90β genes were knocked out in MCF‐7 and T47D cells using CRISPR/Cas9 technology. Immunoblotting analysis was performed with the indicated antibodies. (B–D) WT and HSP90 KO MCF‐7 and T47D cells were treated with DMSO or 1 μM of 17‐AAG, AUY922 or STA‐9090 for 24 h. Total cellular lysates were harvested for immunoblotting analysis. (E,F) Lysates from MCF‐7 and T47D cells were subjected to Co‐IP assays and immunoblotting analysis with the indicated antibodies. (G) MCF‐7 cells were treated with or without 5 μM of trichostatin A for the indicated times. Total cellular lysates were harvested for immunoblotting analysis with the indicated antibodies. (H) MCF‐7 cells were treated with or without 10&nbsp;mM of sodium butyrate (NaBu) for the indicated times. Total cellular lysates were harvested for immunoblotting analysis with the indicated antibodies. (I) MCF‐7 cells were treated with or without 5 mM of NAM for the indicated times. Total cellular lysates were harvested for immunoblotting analysis with the indicated antibodies

We recently reported that MORC2 undergoes lysosomal proteolysis mediated by chaperone‐mediated autophagy (CMA) but not proteasomal degradation.^14^ In CMA, the substrate proteins are specifically recognized by chaperone heat shock protein family A member 8 (HSPA8) and then delivered to lysosome‐associated membrane protein type 2A (LAMP2A) for lysosomal degradation.^38^ In support of our previous results,^14^ knockdown of HSPA8 or LAMP2A by two independent siRNAs increased MORC2 protein levels (Figure S8a,b). Furthermore, reduced MORC2 protein levels in cells treated with HSP90 inhibitors were partially restored following knockdown of HSPA8 or LAMP2A (Figure 2C,D). Immunofluorescent staining showed that, in cells treated with DMSO, MORC2 (red) mainly localized in the nucleus, whereas HSPA8 (green) or LAMP2A (green) mainly localized in the cytoplasm, so the co‐localization of MORC2 with HSPA8 or LAMP2A was rarely seen (Figure 2E, the top row). However, after treatment with HSP90 N‐terminal inhibitors, MORC2 (red) translocated to the cytoplasm, so it was observed an increase in the co‐localization of MORC2 (red) with HSPA8 (green) or LAMP2A (green) in the cytoplasm (Figure 2, 3, 4 rows, yellow colour indicated by the dark blue arrows in the merged images). Quantification of co‐localization images using ImageJ plugin co‐localization finder program^39^, ^40^ also demonstrated that Pearson's correlation coefficient and Manders' co‐localization coefficient, two indicators for protein co‐localization, were increased after treatment with HSP90 N‐terminal inhibitors compared to DMSO control (Figure S8c). Collectively, these results suggest that HSP90 N‐terminal inhibitors induce MORC2 degradation mainly through the CMA pathway.

**FIGURE 4:**
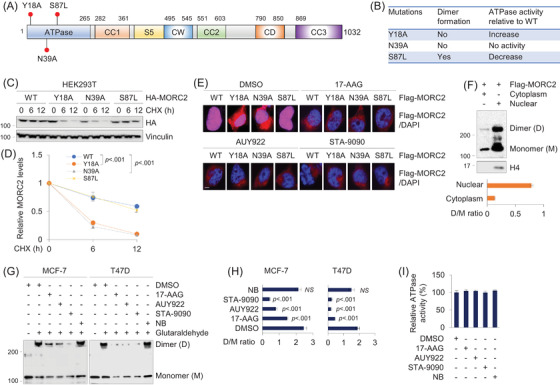
HSP90 N‐terminal inhibitors disrupt MORC2 dimerization in breast cancer cells. (A) Graphic representation of MORC2 domains and site mutagenesis. (B) Summary of the effects of Y18A, N39A and S87L mutations on the dimer formation and ATPase activities of MORC2. (C,D) HEK293T cells were transfected with various HA‐MORC2 plasmids (WT, Y18A, N39A or S87L). After 36 h of transfection, cells were treated with 100 μg/ml of CHX for the indicated times and then subjected to immunoblotting analysis with the indicated antibodies (C). Relative HA‐MORC2 expression levels (HA‐MORC2/Vinculin) are shown in (D). (E) HEK293T cells were transfected with Flag‐MORC2 (WT, Y18A, N39A and S87L). After 24 h of transfection, cells were treated with DMSO or 1 μM of 17‐AAG, AUY922 or STA‐9090 for 24 h, and then subjected to immunofluorescent staining with an anti‐Flag antibody. The nuclei were counterstained with DAPI. Scale bar, 10 μm. (F) HEK293T cells we transfected with Flag‐MORC2. After 48 h of transfection, cells were subjected to cytosol‐nucleus cell fractionation assays, followed by chemical cross‐linking experiment. The quantitative results (dimer/monomer ratio) are shown below. (G,H) MCF‐7 and T47D cells were treated with DMSO or 1 μM of 17‐AAG, AUY922, STA‐9090 or 10 μM of NB for 12 h. Total cellular lysates were harvested for cross‐linking reactions with glutaraldehyde (G). Dimer/monomer ratio is shown (H). (I) Immunoprecipitated Flag‐MORC2 was incubated with 1 μM 17‐AAG, AUY922, STA‐9090 and NB for 1 h at room temperature in the presence of 4 mM ATP. The absorbance at 620 nm was read and analysed

### 3.6 Downregulation of MORC2 by HSP90 N‐terminal inhibitors is independent on HSP90

It is well established that a protein as a HSP90 client relies on two criteria. First, inhibition of HSP90 function must result in a decrease in its protein levels. Second, it must physically interact with HSP90.^41^ In mammalian cells, HSP90α and HSP90β, which are encoded by two individual genes and are present in the cytosol, are collectively called HSP90.^42^ To address whether MORC2 is a novel, canonical client of HSP90, we first knocked out both HSP90α and HSP90β genes in MCF‐7 and T47D cells using CRISPR/Cas9 technology.^29^ Immunoblotting analysis showed that knockout (KO) of HSP90 had no remarkable affect on MORC2 protein levels compared to wild‐type (WT) counterpart (Figure 3A). Furthermore, treatment with three HSP90 N‐terminal inhibitors (17‐AAG, AUY922 and STA‐9090) led to a decrease in MORC2 protein levels in both WT and HSP90 KO cells (Figure 3B–D, respectively).

HSP90 and MORC2 are predominantly localized in the cytoplasm and nucleus, respectively.^8^, ^13^, ^16^, ^42^ To determine the possible binding between MORC2 and HSP90, cellular lysates were subjected to reciprocal immunoprecipitation assays. No evident interaction between HSP90 and MORC2 was observed in these cells (Figure 3E,3F). As a positive control, MORC2 and HSP90 interacted with their known binding partners N‐acetyltransferase 10 (NAT10)^13^ and Akt,^43^ respectively. These results are against the notion that MORC2 is a canonical client of HSP90 chaperone. In support of our results, we noticed that MORC2 is not on the comprehensive list of HSP90 client proteins summarized on the website at the Picard laboratory (https://www.picard.ch/downloads/Hsp90interactors.pdf ).

Post‐translational modifications have been shown to regulate the HSP90 chaperone activity.^44^ In this context, histone deacetylase 6 (HDAC6), a class IIb HDAC, has been shown to deacetylate HSP90 and positively regulate its chaperone function.^44^ Consequently, pharmacological inhibition of HDAC6 blocks HSP90 chaperone activity and degrades client proteins such as Akt. Treatment of MCF‐7 cells with tubastatin A, an inhibitor of HDAC6 (Figure 3G),^45^ resulted in a downregulation of Akt, but not MORC2. As a control, treatment with sodium butyrate, an inactivator of class I and class IIa HDACs, or nicotinamide, a class III HDAC inhibitor, did not regulate the protein levels of both Akt and MORC2 (Figure 3H,I). Together, these results uncover that HSP90 inhibitor‐induced degradation of MORC2 is independent on HSP90.

### 3.7 HSP90 N‐terminal inhibitors disrupt MORC2 dimerization in breast cancer cells

A recent structural and biochemical study has highlighted that ATP binding or dimerization (or both) is crucial for MORC2 biological function.^6^ In particular, mutant MORC2 with an alanine substitution of the key residue tyrosine 18 (Y18A) in the dimer interface is capable for binding and hydrolysing ATP but does not undergo ATP‐dependent dimerization.^6^ In contrast, mutation of the highly conserved ATPase active site at residue asparagine 39 with alanine (N39A) abolishes the capacity of MORC2 to form a dimer or bind or hydrolyse ATP.^6^ In addition, the S87L mutation in the ATP lid exerts reduced ATPase activities^6^, ^18^ and forms a constitutive N‐terminal dimer.^6^ All of those three mutations are localized within its N‐terminal domain (Figure 4A,B). To distinguish the contribution of ATP binding and hydrolysis and dimerization to MORC2 stability, we first compared the turnover rate of three MORC2 mutants (Y18A, N39A and S87L). As displayed in Figure 4C,D, the turnover rate of both N39A and Y18A mutants was faster than WT and S87L mutant MORC2, suggesting that the N‐terminal dimerization, rather than ATP hydrolysis, is critical for MORC2 stability.

To examine whether dimerization of MORC2 occurs dominantly in the nucleus, we transfected HEK293T with Flag‐MORC2 (WT, Y18A, N39A or S87L). After transfection, cells were medicated with DMSO or 17‐AAG, AUY922 or STA‐9090, and then analysed with immunofluorescent staining with an anti‐Flag antibody. The results suggested that, in the absence of HSP90 N‐terminal inhibitors, wild‐type MORC2 and its S87L mutant (red) mainly localized in the nucleus (complete overlapping with DAPI), whereas Y18A and N39A mutant MORC2 localized in both nucleus and cytoplasm. After treatment with HSP90 N‐terminal inhibitors, WT and S87L mutant MORC2 translocated from the nucleus to the cytoplasm, where they are degraded through the CMA pathway (Figure 4E). To validate these results, we transfected Flag‐MORC2 into HEK293T cells, and then performed cytosol‐nucleus cell fractionation assays, followed by chemical cross‐linking experiments. The results showed that nucleus MORC2 mainly existed as dimers, whereas cytosolic MORC2 rarely formed dimers (Figure 4F). Chemical cross‐linking experiments with glutaraldehyde showed that treatment with three HSP90 N‐terminal inhibitors (17‐AAG, AUY922 and STA‐9090), but not C‐terminal inhibitor NB, impaired homodimer formation of MORC2 (Figure 4G,H). To evaluate whether HSP90 N‐terminal inhibitors influence the ATPase activity of MORC2, we carried out a colorimetric ATPase assay using an ATPase/GTPase assay kit that has been used successfully to determine the ATPase activity of MORC2.^6^, ^15^ Three HSP90 N‐terminal inhibitors did not measurably affect the ATPase activity of MORC2 (Figure 4I). Together, these results suggest that HSP90 N‐terminal inhibitors induce MORC2 degradation through, at least in part, attenuating its N‐terminal homodimerization.

### 3.8 HSP90 inhibitor 17‐AAG is effective against MORC2‐driven breast cancer progression

**FIGURE 5:**
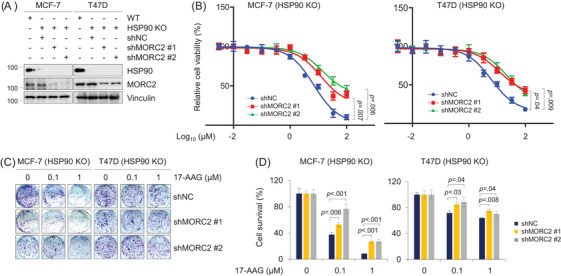
Knockdown of MORC2 reduces the sensitivity of breast cancer cells to 17‐AAG (A) Immunoblotting analysis of HSP90 KO MCF‐7 and T47D cells stably expressing shNC and shMORC2 with the indicated antibodies. The quantitation of immunoblotting bands was performed using ImageJ software. (B) HSP90 KO MCF‐7 and T47D cells stably expressing shNC and shMORC2 were treated with increasing doses of 17‐AAG for 3 days. Cell viability was assessed using CCK‐8 kit. Cell viability (%) was plotted against the log concentration of 17‐AAG. Each dot and error bar on the curves represents mean ± SD (*n* = 3). All experiments were repeated three times. (C,D) HSP90 KO MCF‐7 and T47D cells stably expressing shNC and shMORC2 were treated with 17‐AAG at the indicated doses and subjected to colony formation survival assays (C). Quantitative results are shown in (D)

To examine whether HSP90 inhibitors affect oncogenic functions of MORC2, we first knocked down endogenous MORC2 in HSP90‐KO cells by two shRNAs targeting MORC2 (shMORC2). The expression status of HSP90 and MORC2 was verified (Figure 5A). We next evaluated the sensitivity to HSP90 inhibitor 17‐AAG of these cells, which is effective against breast cancer in preclinical studies and clinical trials.^30^, ^46^, ^47^, ^48^, ^49^ Cell viability assays showed that depletion of MORC2 in HSP90‐KO cells decreased cellular sensitivity to 17‐AAG compared to negative‐control shRNA (shNC) (Figure 5B). Analogous results were obtained from colony formation assays (Figure 5C,D). It is worth noting that these observed effects were not due to HSP90 inhibition, as HSP90 was knocked out in MCF‐7 and T47D cell lines. In agreement with these results, overexpression of MORC2 in Hs578T and MDA‐MB‐231 cells enhanced cellular sensitivity to 17‐AAG as compared with pCDH control (Figure S9).

**FIGURE 6:**
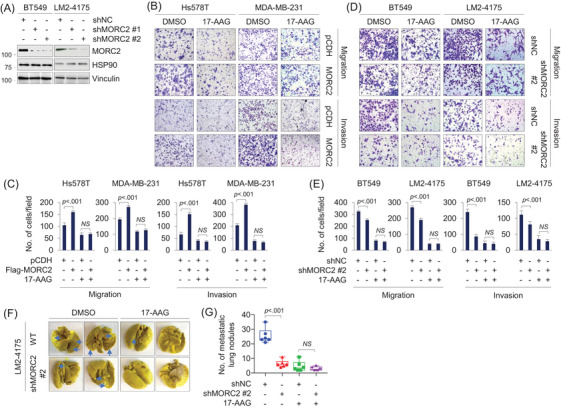
17‐AAG suppresses MORC2‐indcued breast cancer migration, invasion and metastasis. (A) Immunoblotting analysis of BT549 and LM2‐4175 cells stably expressing shNC and shMORC2 with the indicated antibodies. The quantitation of immunoblotting bands was performed using ImageJ software. (B,C) Hs578T and MDA‐MB‐231 cells stably expressing pCDH and Flag‐MORC2 were subjected to Transwell migration and Matrigel invasion assays in the presence or absence of 1 μM of 17‐AAG. Representative images are shown in (C) and corresponding quantitative results are shown in (C). (D,E) BT549 and LM2‐4175 cells stably expressing shNC and shMORC2 were subjected to Transwell migration and Matrigel invasion assays in the presence or absence of 1 μM of 17‐AAG. Representative images are shown in (E) and corresponding quantitative results are shown in (E). (F,G) LM2‐4175 cells stably expressing shNC and shMORC2 were injected into nude mice (*n* = 6) through the tail vein and treated with DMSO or 17‐AAG (60 mg/kg/d). The lungs were harvested after 3 weeks of treatment. Representative images of lung metastasis and corresponding quantitative results of lung nodules are shown in (F) and (G), respectively

Typically, TNBC cells are more aggressive with higher rates of recurrence and metastasis than non‐TNBC ones.^50^ Among TNBC cell lines, MDA‐MB‐231 and Hs578T cells have lower levels of endogenous MORC2 than BT549 and LM2‐4175 cells.^16^ To determine whether 17‐AAG has inhibitory effects on MORC2‐mediated migration and invasion, we expressed Flag‐MORC2 in Hs578T and MDA‐MB‐231 cells (Figure S8a) and knocked down endogenous MORC2 in BT549 and LM2‐4175 cells (Figure 6A). Immunoblotting showed that overexpression or knockdown of MORC2 did not significantly affect HSP90 expression (Figure S9a and Figure 6A, respectively). Transwell assays showed that ectopic expression of MORC2 enhanced the migratory and invasive potential of Hs578T and MDA‐MB‐231 cells (Figure 6B,C), which went along with our previous reports.^16^, ^51^ Furthermore, MORC2‐induced migratory and invasive potential was impaired in cells treated with 17‐AAG, and the noted inhibitory effects of 17‐AAG were more obvious in MORC2‐overexpressing cells than empty vector‐expressing controls (Figure 6B,C). Conversely, shMORC2‐infected LM2‐4175 and BT549 cells exhibited a reduced migratory and invasive phenotype as compared with shNC‐infected cells, and knockdown of MORC2 attenuated the inhibitory effect of 17‐AAG on migratory and invasive potential (Figure 6D,E).

To further confirm these findings in vivo, we injected LM2‐4175 shNC and shMORC2 cells into immunodeficient BALA/c nude mice through tail vein, and then administrated these mice with or without 17‐AAG. The experiment indicated that depletion of MORC2 significantly reduced the number of metastatic tumours in the lungs in vivo as compared with shNC control, and 17‐AAG more effectively reduced lung metastatic potential in mice bearing shNC tumours than in those injected with shMORC2 cells (Figure 6F,G). Immunohistochemical staining of metastatic lung tissues with an anti‐MORC2 antibody showed that MORC2 level was decreased after treatment with 17‐AAG in shNC control group (Figure S10). Together, 17‐AAG is effective against MORC2‐driven breast cancer progression in vitro and in vivo and this effect is related to MORC2 expression levels.

**FIGURE 7:**
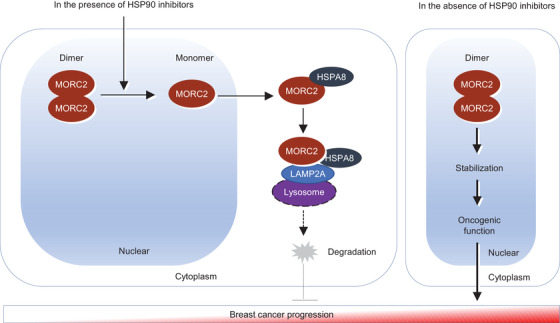
The proposed working model. HSP90 N‐terminal inhibitors target MORC2 for lysosomal degradation through disrupting its N‐terminal homodimerization, and consequently, block MORC2‐mediated breast cancer progression (left). In contrast, in the absence of HSP90 N‐terminal inhibitors, dimer formation leads to an increase of protein stability of MORC2, thus manifesting its oncogenic functions (right)

Collectively, we discovered that HSP90 N‐terminal inhibitors disrupt the homodimer formation of oncogenic ATPase MORC2 to induce its autophagic degradation in a HSP90‐independent manner, and consequently, suppress MORC2‐driven breast cancer progression (Figure 7).

## 4 DISCUSSION

This study first report the small‐molecule‐based antagonists of oncogenic ATPase MORC2. First, three HSP90 N‐terminal inhibitors induce autophagic degradation of MORC2 through disrupting its N‐terminal homodimerization. As both MORC2 and HSP90 utilize a common Bergerat fold to bind ATP,^3^ we hypothesized that some HSP90 inhibitors could target MORC2. In support of this notion, we supply the first evidence that HSP90 N‐terminal inhibitors, but not HSP90 C‐terminal inhibitors and TOP2 inhibitors, destabilized MORC2 in multiple cancer cell lines (Figures 1 and 2 and Figures S1–S6). Recent studies on MORC2 has uncovered that MORC2 contains two putative Lys‐Phe‐Glu‐ArgGln (KFERQ)‐like motifs at N‐terminus and undergoes lysosomal degradation through the CMA pathway.^14^ In support of our previous results,^14^ we further demonstrated that MORC2 downregulation by HSP90 inhibitors was partially restored by inhibition of autophagy but not proteasome (Figure 2A,B). Moreover, HSP90 N‐terminal inhibitors enhanced the co‐localization of MORC2 with HSPA8 or LAMP2A, two key players of the CMA machinery, and knockdown of either HSPA8 or LAMP2A prevented degradation of MORC2 by HSP90 inhibitors (Figure 2C–E). These results suggest that MORC2 degradation by HSP90 inhibitors is dependent on the CMA pathway.

Dimerization of proteins plays a critical role in their stability and activities. Therefore, targeting such dimeric proteins by mAbs or small‐molecule inhibitors could interfere with their biological functions and represent a potential therapeutic approach to treating various diseases. For instance, targeting dimerization of survivin, which was known as a homodimeric member of the inhibitor of apoptosis protein (IAP) family, with inhibitors induces its proteasome‐dependent degradation and effectively inhibits xenograft tumour growth.^52^, ^53^ In addition, inhibition of homodimerization of oncogenic NAC1 protein by small‐molecule compounds leads to proteasomal NAC1 degradation and raises the sensitivity of anticancer agents.^54^ We demonstrated that three HSP90 N‐terminal inhibitors disrupted MORC2 homodimerization without affecting its ATPase activities and induced its lysosomal degradation (Figure 4). Similarly, radicicol, a drug known to inhibit HSP90, blocks the dimerization of the topo VI.^55^

Second, degradation of MORC2 by HSP90 inhibitors is independent of HSP90. HSP90 inhibitors have been shown to degrade nearly 400 clients, including many responsible for tumour initiation and progression.^23^, ^56^ Clients of HSP90 are strictly dependent on HSP90 for their maturation and function and defined by two standards. It must physically bind with HSP90, and inhibiting HSP90 must lead to a decrease in its protein levels.^41^ Here, we identified the first oncoprotein that is degraded by HSP90 inhibitors in a HSP90‐independent manner based on the following evidence (Figure 3). First, there is no evident binding between MORC2 and HSP90. Second, knockout of HSP90 or inactivation of chaperone function of HSP90 by pharmacological inhibition of HDAC6^44^, ^57^ does not affect the basal levels of MORC2 (Figure 3). Third, the noted downregulation of MORC2 by HSP90 N‐terminal inhibitors are also observed in HSP90‐deficient cells. In support of our results, MORC2 is not on the documented comprehensive list of HSP90 client proteins. These results are against the notion that MORC2 is a canonical client of HSP90.

Third, HSP90 N‐terminal inhibitor 17‐AAG effectively blocks MORC2‐mediated breast cancer progression, and this effect is independent on HSP90. Multiple preclinical studies and clinical trials have documented potent anticancer effects of HSP90 inhibitors against breast cancer as monotherapy or in combination. In this context, 17‐AAG displays strong antitumour efficacy in all three molecular subtypes of breast cancer cells, and sensitizes cancer cells to cytotoxic chemotherapeutic agents (such as taxol), radiation, and other targeted therapeutic agents such as HDAC inhibitors, mTOR inhibitor rapamycin and HER2‐targeting antibody trastuzumab.^30^, ^46^, ^47^, ^48^, ^49^ Phase I/II clinical trials also demonstrated that 17‐AAG combinating with trastuzumab has remarkable anticancer activity in HER2‐positive, metastatic breast cancer.^48^, ^49^ In our study, we discovered that MORC2‐expressing breast cancer cells had greater sensitivity to 17‐AAG both in vitro and in vivo (Figure 5 and 6, and Figure S10). Furthermore, this phenomenon is independent on HSP90 inhibition. These results may expand the therapeutic utility of HSP90 inhibitors in MORC2‐expressing tumours, even those with low or absent HSP90 expression.

In summary, findings presented here suggest that HSP90 N‐terminal inhibitors induce autophagic degradation of MORC2 in a HSP90‐independent manner through disrupting its homodimerization and are effective against MORC2‐expressing breast tumours irrespective of HSP90 expression status. Future studies are needed to define whether HSP90 N‐terminal inhibitors in combination with conventional endocrinal therapy drugs or DNA‐damaging chemotherapeutic agents or radiotherapy have synergistic effects on MORC2‐expressing cancers.

## CONFLICT OF INTEREST

The authors declare that they have no conflict of interest.

## Supporting information



Supplementary material
