# Supplementary material for: HSP90 N‐terminal inhibitors target oncoprotein MORC2 for autophagic degradation and suppress MORC2‐driven breast cancer progression
Source: Clin Transl Med. 2022 May 6;12(5):e825. doi: 10.1002/ctm2.825 (PMC9076019; doi:10.1002/ctm2.825)
Supplement: Supplementary file 1 — Supplementary material [file CTM2-12-e825-s001.docx]

**Supporting Information for**

Yang F, et al. HSP90 N-terminal inhibitors promote autophagic degradation of oncoprotein MORC2 and suppress MORC2-driven breast cancer metastasis

**The Supplementary Information includes**

1. Supplementary Figures S1-S10

2. Supplementary Figure legends

3. Supplementary Tables S1-S8

**Supplementary Figures and Figure legends**

**
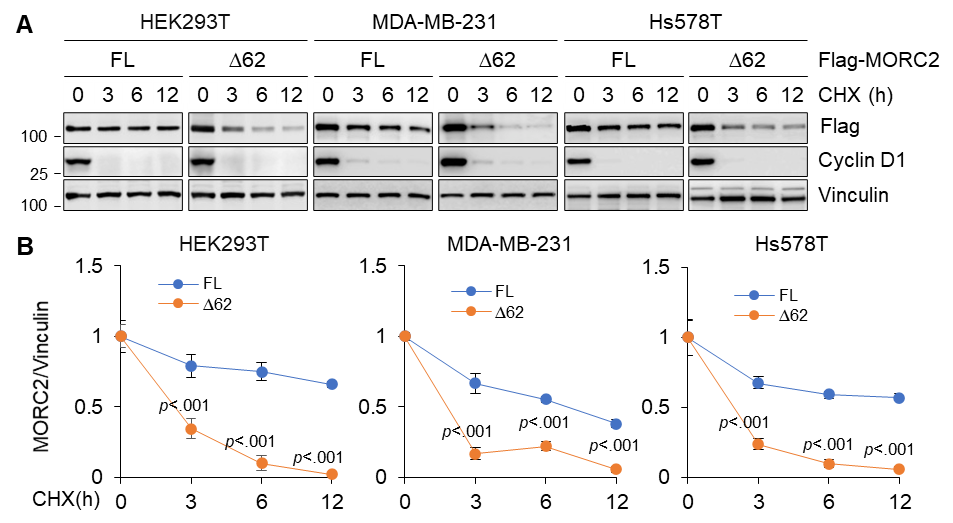
**

**Fig. S1. The intact N-terminal ATPase module of MORC2 is crucial for its stability**

**(A-B**) Cells were transfected with full-length or ∆62 mutant Flag-MORC2. After 48 h of transfection, cells were treated with 100 µg/mL of CHX for the indicated times, and total cellular lysates were subjected to immunoblotting analysis with the indicated antibodies (**A**). The quantitation of immunobloting bands was performed using ImageJ software. The relative expression levels of Flag-MORC2 are shown in **B**.

**
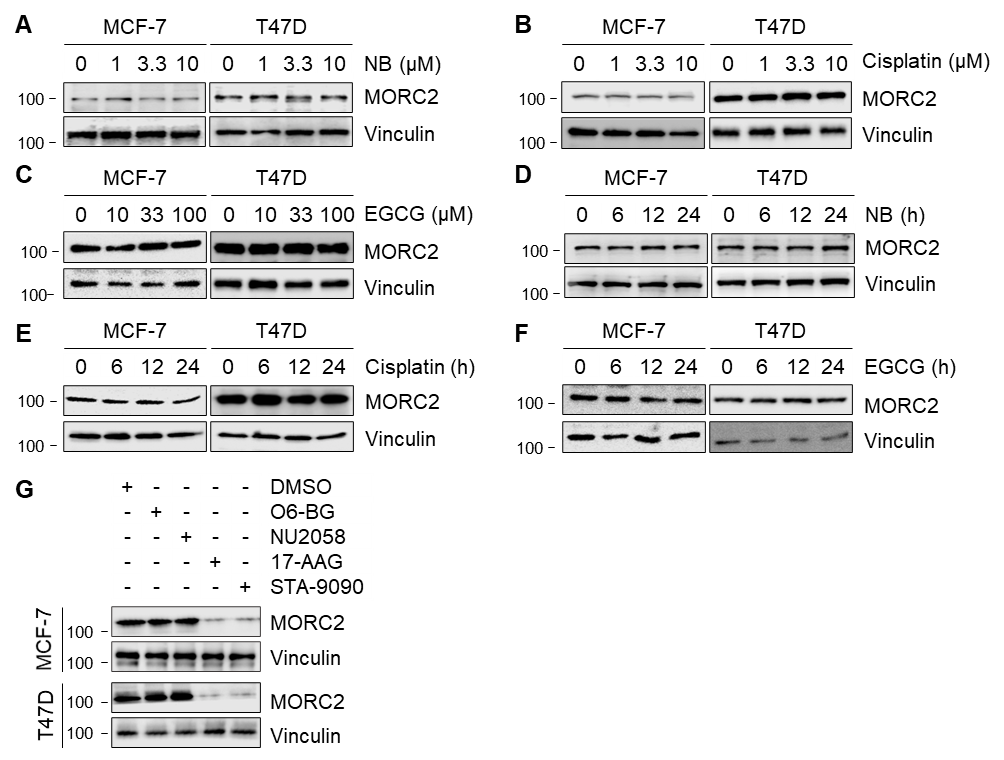
**

**Fig. S2. HSP90 C-terminal inhibitors and TOP2 inhibitors have no significant effects on MORC2 expression levels**

(**A-C**) MCF-7 and T47D cells were treated with or without NB (A), Cisplatin (B), EGCG (C) at the indicated doses for 24 h. Total cellular lysates were harvested for immunoblotting analysis with the indicated antibodies. (**D-F**) MCF-7 and T47D cells were treated with or without 10 μM NB (D), 10 μM cisplatin (E), or 100 μM EGCG (F) for the indicated times. Total cellular lysates were harvested for immunoblotting analysis with the indicated antibodies. (**G**) MCF-7 and T47D cells were treated with or without 300 μM O6-BG, 300 μM NU2058, 1 μM 17-AAG or 1 μM STA-9090 for 24 h. Total cellular lysates were harvested for immunoblotting analysis with the indicated antibodies.


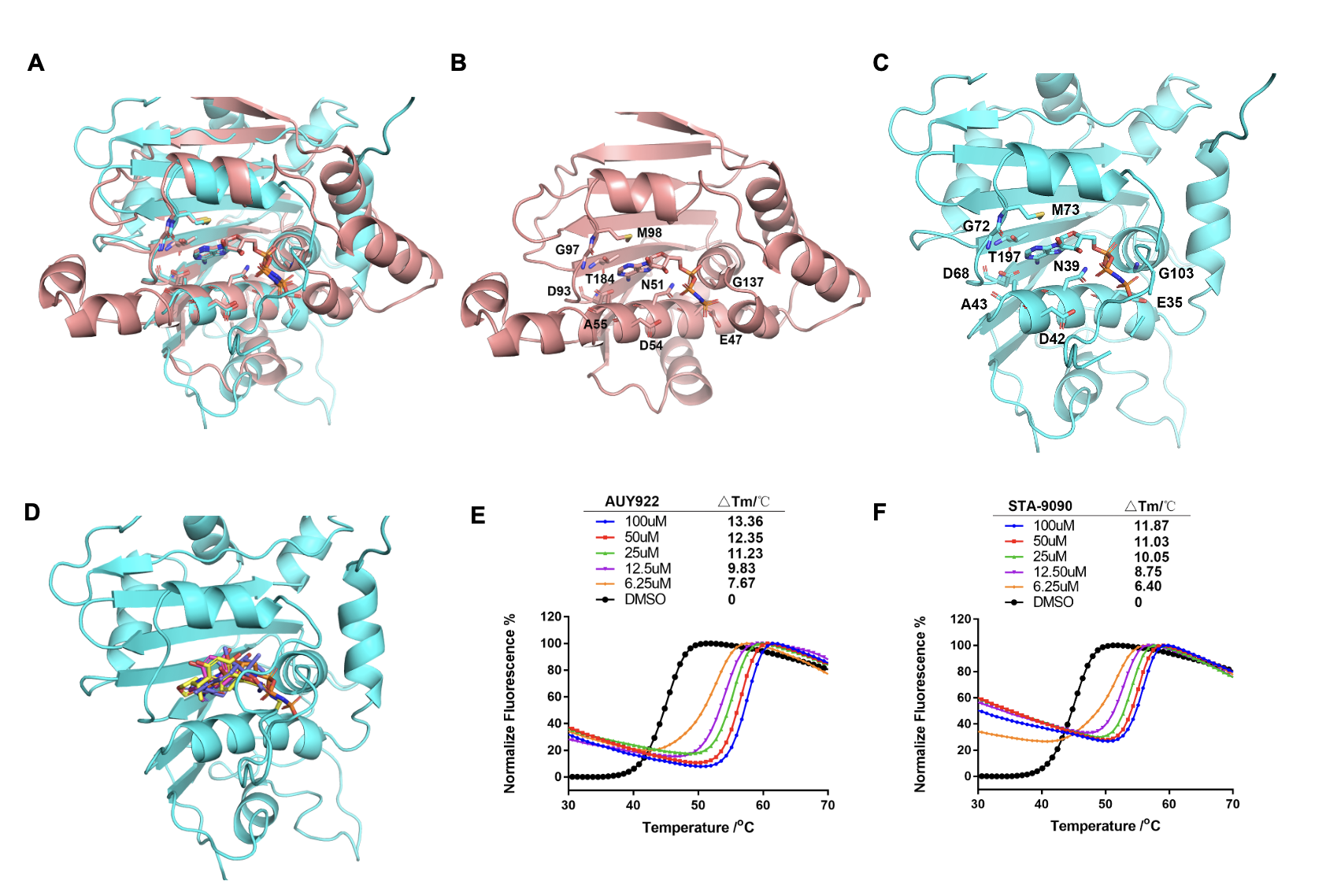


**Fig. S3. HSP90 N-terminal inhibitors bind directly to the N-terminus of MORC2**

(A) Comparison of the ATP binding pocket of Hsp90 N-terminal (salmon) with MORC2 N-terminal (cyan). (B-C) The details of amino acids within the ATP binding pocket of Hsp90 N-terminal (salmon) that have homology to MORC2 N-terminal (cyan). These amino acids are shown as stick. (D) Overlay of the states of 17-AAG (purple), AUY922 (yellow), STA-9090 (magenta), and ATP (orange) in the ATP binding pocket of MORC2 N-terminal (cyan). (E-F) Changes in thermodynamic stability of MORC2 N-terminal upon binding with AUY922 or STA-9090. The △Tm was calculated as the difference vs. the DMSO control sample.

**
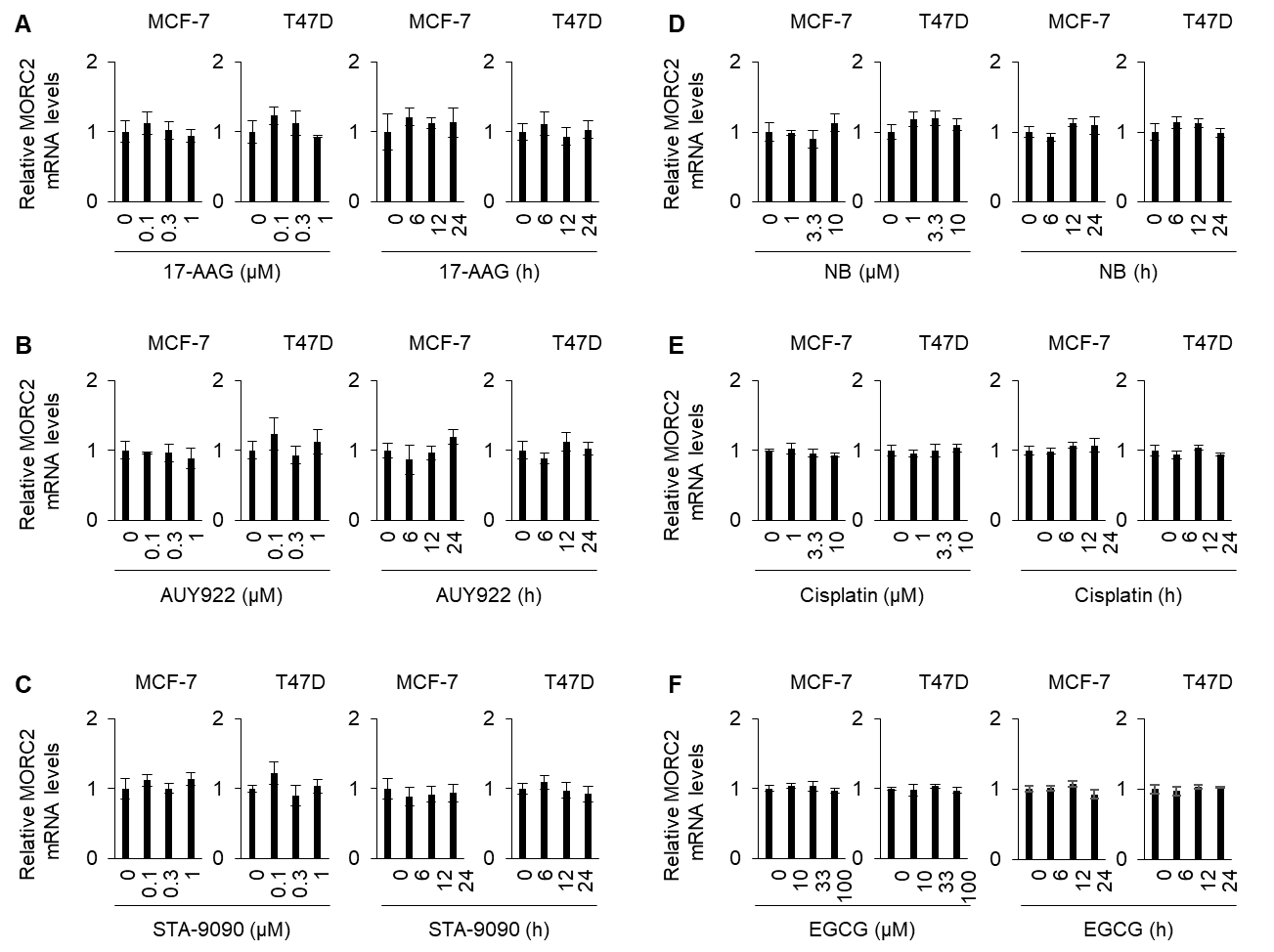
**

**Fig. S4. HSP90 N-terminal and C-terminal inhibitors do not affect the mRNA levels of MORC2**

(**A**) MCF-7 and T47D cells were treated with or without 17-AAG at the indicated doses for 24 h or 1 µM 17-AAG for indicated times, and then harvested for qPCR analysis. (**B**) MCF-7 and T47D cells were treated with or without AUY922 at the indicated doses for 24 h or 1 µM 17-AAG for indicated times, and then harvested for qPCR analysis. (**C**) MCF-7 and T47D cells were treated with or without STA-9090 at the indicated doses for 24 h or 1 µM 17-AAG for indicated times, and then harvested for qPCR analysis. (**D**) MCF-7 and T47D cells were treated with or without NB at the indicated doses for 24 h or 10 µM NB for indicated times, and then harvested for qPCR analysis. (**E**) MCF-7 and T47D cells were treated with or without cisplatin at the indicated doses for 24 h or 10 µM cisplatin for indicated times, and then harvested for qPCR analysis. (**F**) MCF-7 and T47D cells were treated with or without EGCG at the indicated doses for 24 h or 100 µM EGCG for indicated times, and then harvested for qPCR analysis.

**
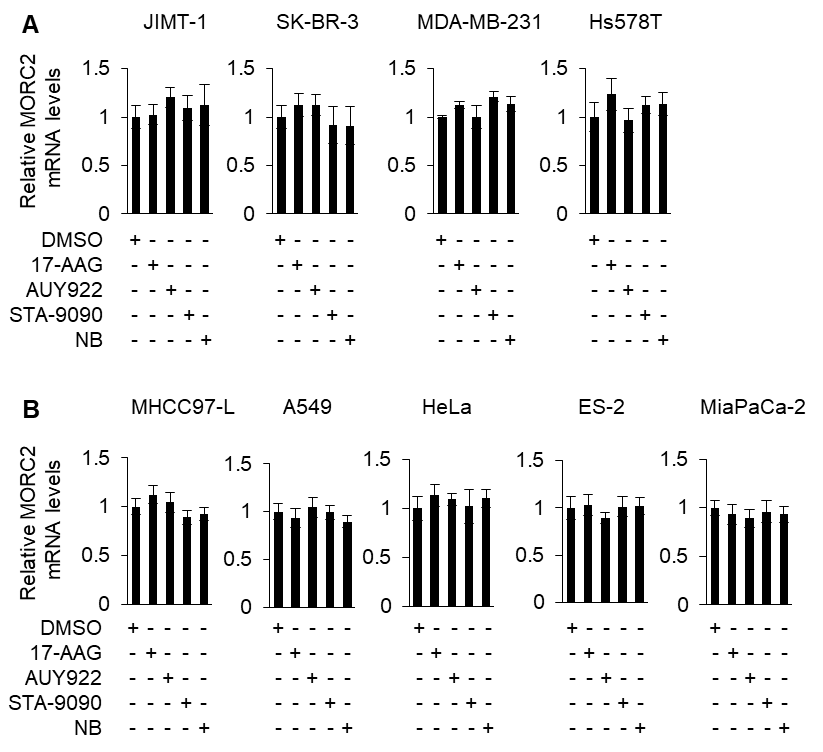
**

**Fig. S5. HSP90 N-terminal and C-terminal inhibitors do not affect the mRNA levels of MORC2**

(**A-B**) Four breast cancer cell lines (A) and five other cancer cell lines (B) were treated with DMSO or 1 µM of 17-AAG, AUY922, STA-909017-AAG, or 10 µM NB for 24 h and then harvested for qPCR analysis.

**
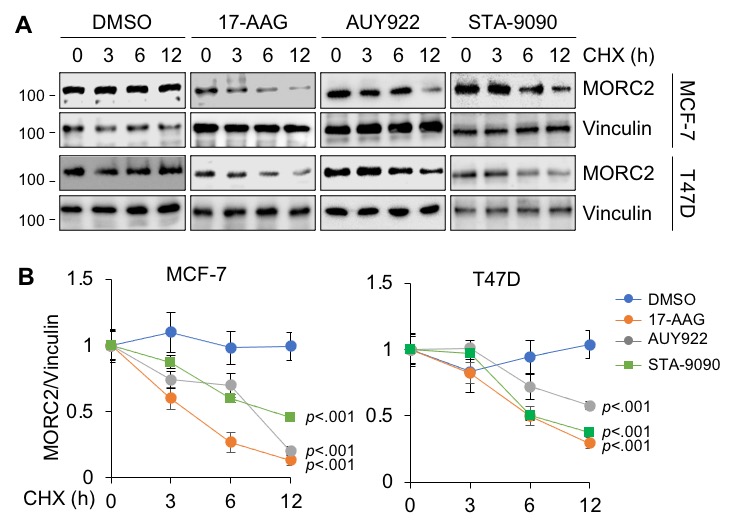
**

**Fig. S6. HSP90 N-terminal inhibitors shorten the half-life of MORC2 protein**

(**A**) MCF-7 and T47D cells were treated with 100 µg/mL of CHX alone or in combination with 1 µM 17-AAG, AUY922, STA-9090 for the indicated times.Total cellular lysates were harvested for immunoblotting analysis with the indicated antibodies. (**B**) Protein band densities were quantified using ImageJ program. Quantitative results of relative expression levels of MORC2 (MORC2/vinculin) are shown, respectively.

**
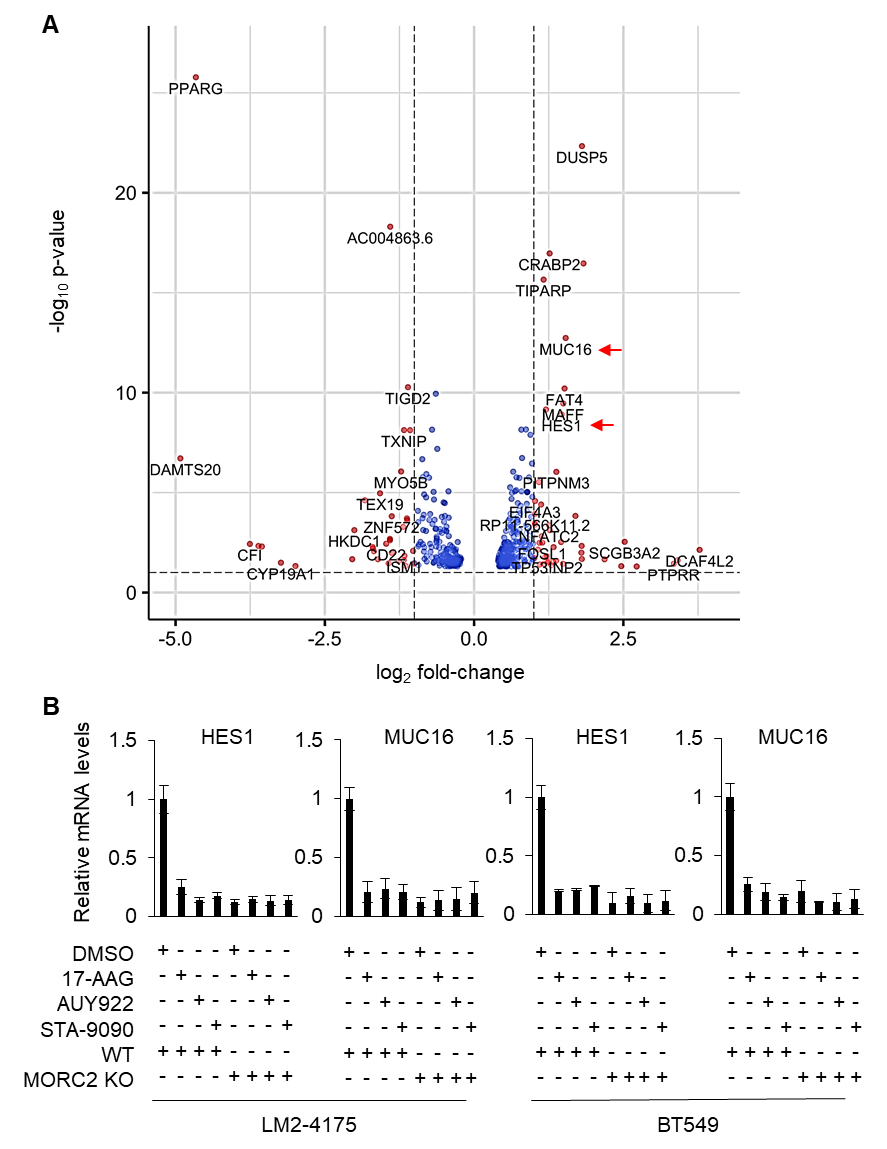
**

**Fig. S7. The effects of HSP90 N-terminal inhibitors on the expression of downstream target genes of MORC2**

(**A**) Volcano plot for differentially expressed genes between wild-type and MORC2 KO HeLa cells. Red arrows indicate two genes that were selected for further validation by qPCR using wild-type and MORC2-depleted LM2-4175 and BT549 cells after treatment with HSP90 N-terminal inhibitors. (**B**) Wild-type (WT) and MORC2-knockout (KO) BT549 and LM2-4175 cells were treated with 1 µM of 17-AAG, AUY922, or STA-9090 for 24 h and then harvested for qPCR analysis.

**
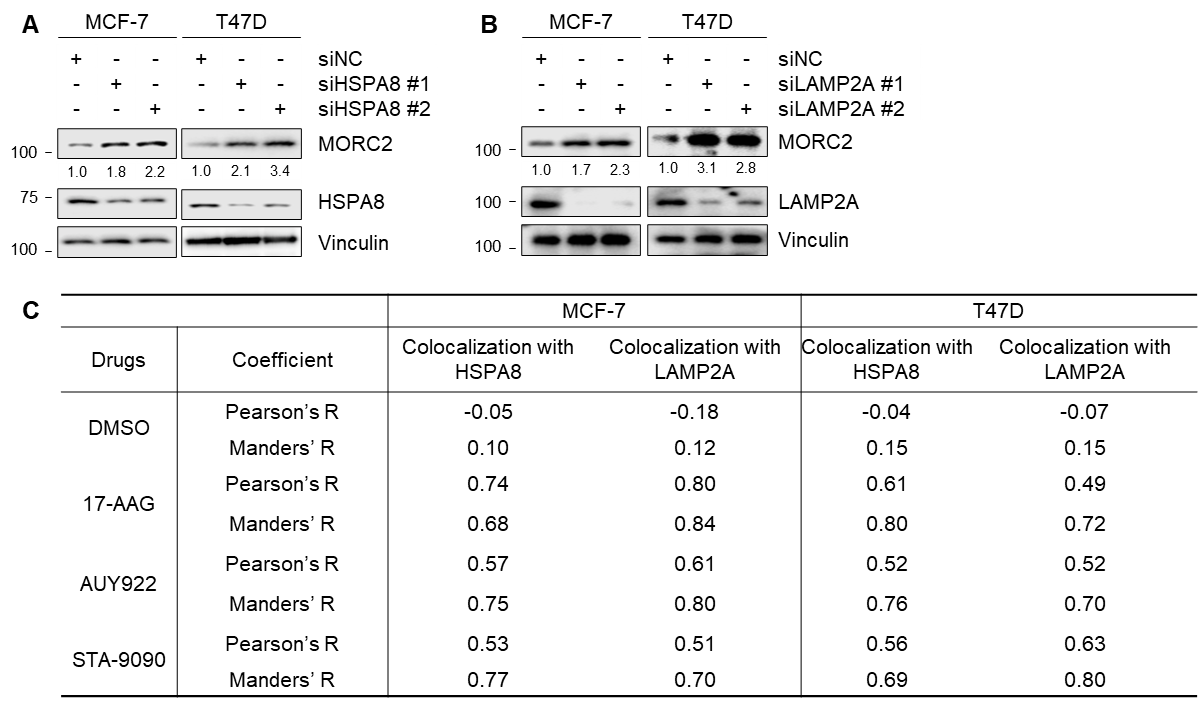
**

**Fig. S8. Knockdown of HSPA8 or LAMP2A results in an upregulation of MORC2 protein levels**

(**A-B**) MCF-7 and T47D cells were transfected with control siRNA (siNC) or siRNAs targeting HSPA8 (A) or LAMP2A (B). After 48 h of transfection, total cellular lysates were harvested for immunoblotting analysis with indicated antibodies. The quantitation of immunobloting bands was performed using ImageJ software. **(C)** Quantification of colocalization images using Image-J plug-in colocalization finder manager. Pearson’s correlation coefficients and Manders’ colocalization coefficients are indicators for protein colocalization ^3^.


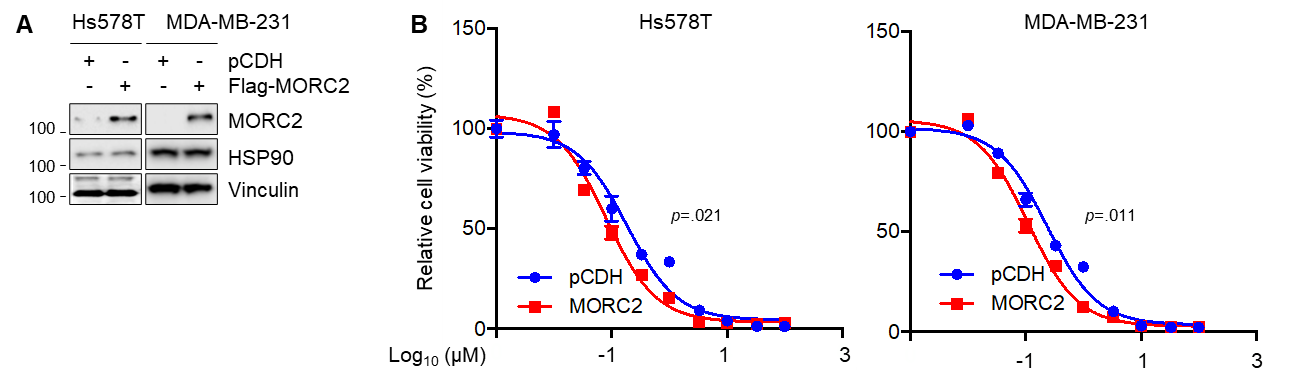


**Fig. S9. The effects of MORC2 overexpression on cellular sensitivity to 17-AAG**

(**A**) Immunoblotting analysis of Hs578T and MDA-MB-231 cells stably expressing pCDH and Flag-MORC2 with the indicated antibodies. The quantitation of immunobloting bands was performed using ImageJ software. (**B**) Hs578T and MDA-MB-231 cells stably expressing pCDH and Flag-MORC2 were subjected to IC50 assays after treated with increasing doses of 17-AAG for 3 days. Cell viability (%) was plotted against the log concentration of 17-AAG. Each dot and error bar on the curves represents mean ± SD (n=3). All experiments were repeated three times.


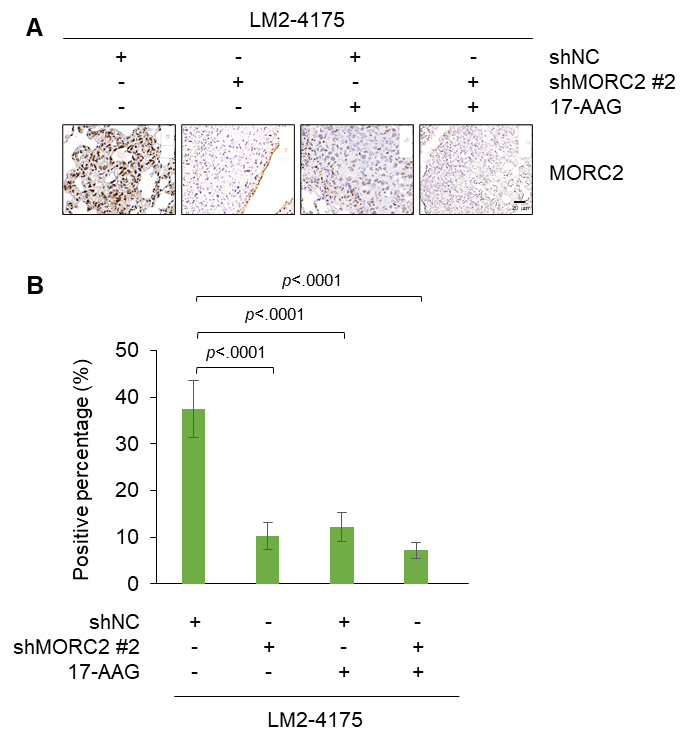


**Fig. S10. HSP90 inhibitor 17-AAG promotes MORC2 degradation *in vivo***

(**A-B**) Representative immunochemical staining images (A) and quantification results (B) of MORC2 expression in metastatic lung specimens from mice treated with DMSO or 60 mg/kg/day 17-AAG for 3 weeks.

**Supplementary Tables**

**Table S1. Chemical reagents used in this study**

| Chemical reagents | Vendors | Catalog # | Working concentration |
| --- | --- | --- | --- |
| Tanespimycin (17-AAG) | Selleck | S1141 | 1 μM |
| Luminespib (AUY922) | Selleck | S1069 | 1 μM |
| Ganetespib (STA-9090) | Selleck | S1159 | 1 μM |
| Novobiocin (NB) | Selleck | S2492 | 10 μM |
| Cisplatin | Selleck | S1166 | 10 μM |
| (-)-Epigallocatechin Gallate (EGCG) | Selleck | S2250 | 100 μM |
| O6-benzylguanine (O6-BG) | Selleck | S3658 | 300 μM |
| NU2058 | Selleck | S5316 | 300 μM |
| Trichostatin A | Selleck | S1045 | 5 µM |
| Sodium butyrate (NaBu) | Selleck | S1999 | 10 mM |
| Nicotinamide (NAM) | Sigma | N0636-100G | 5 mM |
| Cycloheximide | CST | 2112S | 100 μg/ml |
| MG-132 | Selleck | S2619 | 10 μM |
| Bafilomycin A1 (Baf A1) | Selleck | S1413 | 100 nM |

**Table S2. Primers used for molecular cloning of expression vectors**

| Expression vectors | Primers | Sequences |
| --- | --- | --- |
| Flag-MORC2 | Forward | ACCTCCATAGAAGATTCTAGAGCCACCATGGCTTTCACAAATTACAGCAGTC |
|  | Reverse | GATCCATTTAAATTCGAATTCCTTATCGTCGTCATCCTTGTAATCGTCCCCCTTGGTGATGAGGTCCT |
| Flag-MORC2 ∆62 | Forward | ACCTCCATAGAAGATTCTAGAGCCACCATGCTTTGCTTTTTGGATGATGGAG |
|  | Reverse | GATCCATTTAAATTCGAATTCCTTATCGTCGTCATCCTTGTAATCGTCCCCCTTGGTGATGAGGTCCT |
| HA-MORC2 | Forward | ACCTCCATAGAAGATTCTAGAGCCACCATGGCTTTCACAAATTACAGCAGTC |
|  | Reverse | GATCCATTTAAATTCGAATTCTTAAGCGTAATCTGGAACATCGTATGGGTAGTCCCCCTTGGTGATGAGGTC |
| HA-MORC2 Y18A | Forward | CTCAGCTAACCTTTGAAGCTCTGCACACAAATTCAAC |
|  | Reverse | GTTGAATTTGTGTGCAGAGCTTCAAAGGTTAGCTGAG |
| HA-MORC2 N39A | Forward | GCTGAACTGGTTGATGCTGCAAGAGATGCTG |
|  | Reverse | CAGCATCTCTTGCAGCATCAACCAGTTCAGC |
| HA-MORC2 S87L | Forward | CAGTTTGGGAAGTTGGCCAAGCGAACAC |
|  | Reverse | GTGTTCGCTTGGCCAACTTCCCAAACTG |

**Table S3. siRNAs targeting sequences for HSPA8 and LAMP2A**

| Gene | Sources | Sequences |
| --- | --- | --- |
| siHSPA8 #1 | GenePharma | 5'- GCUGUUGUCCAGUCUGAUATT-3' |
| siHSPA8 #2 | GenePharma | 5'- CCAAGCAGACACAGACCUUTT-3' |
| siLAMP2A #1 | GenePharma | 5'- CGCUAUGAAACUACAAAUATT-3' |
| siLAMP2A #2 | GenePharma | 5'- GCUCUACUUAGACUCAAUATT-3' |

**Table S4. sgRNA sequences targeting HSP90**

| Gene | Primers | Sequences |
| --- | --- | --- |
| sgHSP90 #1 | Forward | CACCGACGATGATGAGCAGTACGCT |
|  | Reverse | CTGCTACTACTCGTCATGCGACAAA |
| sgHSP90 #2 | Forward | CACCGCTGCTCATCATCGTTATGTT |
|  | Reverse | CGACGAGTAGTAGCAATACAACAAA |

**Table S5. Information for primary antibodies used in this study**

| Antibodies | Vendors | Cat# | Species | WB | IP | IF | IHC |
| --- | --- | --- | --- | --- | --- | --- | --- |
| HSPA8 | Abcam | ab51052 | RabMab | √ |  | √ |  |
| LAMP2A | Abcam | ab18528 | RabMab | √ |  | √ |  |
| MORC2 | Bethyl | A300-149A | RabMab | √ | √ |  |  |
| MORC2 | Novus | NBP1-89295 | RabMab |  |  |  | √ |
| HA | CST | 3724 | RabMab | √ | √ |  |  |
| Cyclin D1 | CST | 2978 | RabMab | √ |  |  | √ |
| AKT | CST | 4691 | RabMab | √ |  |  | √ |
| HSP90 | Abcam | ab203126 | RabMab | √ | √ |  |  |
| LC3A/B | CST | 12741 | RabMab | √ |  |  |  |
| CDK4 | CST | 12790S | RabMab | √ |  |  |  |
| Flag | Sigma | F3165 | MouMab | √ | √ | √ |  |
| H4 | Abcam | ab40888 | RabMab | √ |  |  |  |
| Vinculin | Sigma | V9131 | MouMab | √ |  |  |  |

*Notes: RabMab, rabbit monoclonal; MouMab, mouse monoclonal.*

**Table S6. Primers used for quantitative real-time PCR analysis**

| Genes | Primers | Sequences |
| --- | --- | --- |
| MORC2 | Forward | TATGCCGCTGTGCTCTAT |
|  | Reverse | TTCTTCACCTCCTGCTCC |
| MUC16 | Forward | CCAGTCCTACATCTTCGGTTGT |
|  | Reverse | AGGGTAGTTCCTAGAGGGAGTT |
| HES1 | Forward | TCAACACGACACCGGATAAAC |
|  | Reverse | GCCGCGAGCTATCTTTCTTCA |
| ꞵ-actin | Forward | GGACTTCGAGCAAGAGATGG |
|  | Reverse | AGCACTGTGTTGGCGTACAG |

**Table S7. HSP90 inhibitors used in this study**

| HSP90 inhibitors | Drugs | Clinical trials/use in breast cancer | References |
| --- | --- | --- | --- |
| N-terminal inhibitor | 17-AAG (Tanespimycin) | Phase 2: HER2-positive metastatic breast cancer | ^1^ |
|  |  | Phase 1: HER2-positive breast cancer | ^2^ |
|  |  | Phase 2: Advanced breast cancer | ^3^ |
|  | STA-9090 (Ganetespib) | Phase 1: HER2-positive metastatic breast cancer | ^4^ |
|  |  | Phase 2: Metastatic breast cancer | ^5^ |
|  | AUY922 (Luminespib) | Phase 2: Metastatic breast cancer | ^6^ |
| C-terminal inhibitors | NB (Novobiocin) | Phase 1: Metastatic breast cancer | ^7^ |
|  | EGCG | Phase 1: Adjuvant radiotherapy | ^8^ |
|  | Cisplatin | Clinical use | |

**Table S8. The working concentrations of HSP90 inhibitors according to the literatures**

| HSP90 inhibitors | | IC50 | Breast cancer cell lines | References |
| --- | --- | --- | --- | --- |
| N-terminal | 17-AAG | 58 nM | MCF-7 | ^9^ |
|  | STA-9090 | 100 nM | MDA-MB-231 | ^10^ |
|  | AUY-922 | 20-40 nM | MCF-7 | ^11^ |
| C-terminal | NB | 205.1 μM | MDA-MB-231 | ^12^ |
|  | Cisplatin | 8.897 μM | MCF-7 | ^13^ |
|  | EGCG | 5~40 μM | MDA-MB-231 | ^14^ |

**References**

1. Modi S, Stopeck A, Linden H, et al. HSP90 inhibition is effective in breast cancer: a phase II trial of tanespimycin (17-AAG) plus trastuzumab in patients with HER2-positive metastatic breast cancer progressing on trastuzumab. *Clin Cancer Res*. 2011;17(15):5132-9. doi:10.1158/1078-0432.CCR-11-0072

2. Modi S, Stopeck AT, Gordon MS, et al. Combination of trastuzumab and tanespimycin (17-AAG, KOS-953) is safe and active in trastuzumab-refractory HER-2 overexpressing breast cancer: a phase I dose-escalation study. *J Clin Oncol*. 2007;25(34):5410-7. doi:10.1200/JCO.2007.11.7960

3. Gartner EM, Silverman P, Simon M, et al. A phase II study of 17-allylamino-17-demethoxygeldanamycin in metastatic or locally advanced, unresectable breast cancer. *Breast Cancer Res Treat.* 2012;131(3):933-7. doi:10.1007/s10549-011-1866-7

4. Jhaveri K, Wang R, Teplinsky E, et al. A phase I trial of ganetespib in combination with paclitaxel and trastuzumab in patients with human epidermal growth factor receptor-2 (HER2)-positive metastatic breast cancer. *Breast Cancer Res.*2017;19(1):89. doi:10.1186/s13058-017-0879-5

5. Jhaveri K, Chandarlapaty S, Lake D, et al. A phase II open-label study of ganetespib, a novel heat shock protein 90 inhibitor for patients with metastatic breast cancer. *Clin Breast Cancer*. 2014; 14(3):154-60. doi:10.1016/j.clbc.2013.12.012

6. Kong A, Rea D, Ahmed S, et al. Phase 1B/2 study of the HSP90 inhibitor AUY922 plus trastuzumab in metastatic HER2-positive breast cancer patients who have progressed on trastuzumab-based regimen. *Oncotarget*. 2016;7(25):37680-37692. doi:10.18632/oncotarget.8974

7. Kennedy MJ, Armstrong DK, Huelskamp AM, et al. Phase I and pharmacologic study of the alkylating agent modulator novobiocin in combination with high-dose chemotherapy for the treatment of metastatic breast cancer. *J Clin Oncol*. 1995;13(5):1136-43. doi:10.1200/JCO.1995.13.5.1136

8. Zhao H, Zhu W, Jia L, et al. Phase I study of topical epigallocatechin-3-gallate (EGCG) in patients with breast cancer receiving adjuvant radiotherapy. Br J Radiol. 2016;89(1058): 20150665. doi:10.1259/bjr.20150665

9. Menzella H, Tran T, Carney J, et al. Potent non-benzoquinone ansamycin heat shock protein 90 inhibitors from genetic engineering of Streptomyces hygroscopicus. *J Med Chem*. 2009;52(6):1518-21. doi:10.1021/jm900012a

10. Proia D, Zhang C, Sequeira M, et al. Preclinical activity profile and therapeutic efficacy of the HSP90 inhibitor ganetespib in triple-negative breast cancer. *Clin Cancer Res*. 2014;20(2):413-24. doi:10.1158/1078-0432.Ccr-13-2166

11. Mohammadian M, Feizollahzadeh S, Mahmoudi R, Toofani Milani A, Rezapour-Firouzi S, Karimi Douna B. Hsp90 Inhibitor; NVP-AUY922 in Combination with Doxorubicin Induces Apoptosis and Downregulates VEGF in MCF-7 Breast Cancer Cell Line. *Asian Pac J Cancer Prev.*  2020;21(6):1773-1778. doi:10.31557/apjcp.2020.21.6.1773

12. Mbaba M, de la Mare J, Sterrenberg J, et al. Novobiocin-ferrocene conjugates possessing anticancer and antiplasmodial activity independent of HSP90 inhibition. *J Biol Inorg Chem*. 2019;24(2):139-149. doi:10.1007/s00775-018-1634-9

13. Hashem H, Amr A, Nossier E, Elsayed E, Azmy E. Synthesis, Antimicrobial Activity and Molecular Docking of Novel Thiourea Derivatives Tagged with Thiadiazole, Imidazole and Triazine Moieties as Potential DNA Gyrase and Topoisomerase IV Inhibitors. *Molecules*. 2020;25(12)doi:10.3390/molecules25122766

14. Chen D, Pamu S, Cui Q, Chan T, Dou Q. Novel epigallocatechin gallate (EGCG) analogs activate AMP-activated protein kinase pathway and target cancer stem cells. *Bioorg Med Chem.*

2012;20(9):3031-7. doi:10.1016/j.bmc.2012.03.002
